# Clinical Updates for Gastrointestinal Malignancies

**DOI:** 10.3390/jpm13091424

**Published:** 2023-09-21

**Authors:** Carmelo Laface, Riccardo Memeo

**Affiliations:** 1 Medical Oncology, Dario Camberlingo Hospital, 72021 Francavilla Fontana, Italy; 2 Unit of Hepato-Pancreatic-Biliary Surgery, “F. Miulli” General Regional Hospital, 70021 Acquaviva Delle Fonti, Italy

Gastrointestinal (GI) cancers include hepatobiliary tumors, pancreatic cancer (PC), neuroendocrine tumors of the gastrointestinal tract, small bowel carcinomas, gastric cancer (GC), anal canal cancer, primary gastric and intestinal lymphomas, gastrointestinal stromal tumors (GISTs) and the most frequent colorectal cancer (CRC). Globally, they correspond to almost 25% and 35% of all cancer-related diagnoses and deaths in the world, respectively [1]. Despite these relevant epidemiological data, screening strategies are only available for colorectal, gastric, and esophageal cancers [2]. Therefore, GI cancers are often diagnosed at an advanced stage when a cure is not possible and therapeutic options are limited. In addition, about 50% of patients with a diagnosis of early stage GI cancer, undergoneto a potentially curative treatment, will develop a recurrence of the disease [3]. These conditions explain the poor prognosis of these patients. In the last decade, new biotechnologies have been developed favoring cancer research and giving a great contribution to the discovery and validation of novel molecular biomarkers predictive of drug response (efficacy/toxicity) and prognosis in several GI cancers such as CRC, GC, biliary cancer, and GISTs. These biomarkers usually correspond to somatic alterations in cancer cells and are commonly employed in clinical practice. Examples are specific mutations in the DNA Mismatch Repair (MMR), neurotrophic tropomyosin-receptor kinase (NTRK), KIT, RAS, BRAF, HER-2, FGFR, and IDH-1 gene mutations. However, tumors have a high level of heterogeneity, both among patients and tumor sites in the same patient, and the known biomarkers regard only small proportions of GI cancer patients.

Hence, there is a need to search for other prognostic biomarkers able to define those patients with a higher risk of disease progression and for predictive biomarkers, both to avoid the administration of inactive drugs to resistant patients and to define the best personalized therapy. In this regard, various potential tissue or bio-fluid biomarkers are currently proposed with relevant data [4].

As regards CRC, biomarkers predictive of drug response for targeted agents (RAS wild-type for anti-EGFR and Microsatellite Instability-High (MSI-H) status for anti-programmed cell death 1 (anti-PD-1) monoclonal antibodies, NTRK gene fusions, BRAF V600E and KRAS G12C mutations) are employed only in the advanced stage [5]. The knowledge of the MSI-H status provides various clinical information. First, it favors the inherited predisposition to GI cancers due to an impaired MMR system and is associated with a better prognosis for early stage CRC patients. In addition, MSI status is correlated with no benefit from adjuvant chemotherapy for low-risk stage II CRC patients [6]. Treatment options in CRC are associated with the cancer setting, differing from metastatic and non-metastatic stages. In detail, patients with stage I and low-risk stage II are candidates for surgery alone while pharmacological therapy plus surgery is the treatment strategy for patients with high-risk stage II, stage III, and stage IV (oligometastatic disease). However, despite the curative treatments, almost 30% of early stage CRC patients will develop a recurrence of the disease [7].

Pancreatic cancer (PC) has a severe prognosis due to late diagnosis and poor therapeutic options [8,9,10,11,12]. Hence, it is importance to identify specific biomarkers to predict the response to therapies [13]. Several data suggest a potential role of high tumor mutational burden (TMB) as a predictive biomarker of immunotherapy response for PC, although a cut-off to indicate a TMB as “high” (TMB-H) has not been defined yet. However, PC with high TMB represents only 1.1% of cases [14]. In almost 60% of cases, TMB-H PC is correlated to MMR deficiency (dMMR) or MSI-H [13]. Clinical data show that PC patients with high-TMB and MSI-H/dMMR experienced interesting results when treated with anti-PD-1 antibodies although poor compared to other tumors; moreover, these data derive from very small patient populations [13]. Currently, the presence of MSI-H/dMMR is the only factor proven to predict the response to immune-checkpoint inhibitors (ICIs) for PC, but it regards only 3% of PC patients [15,16]. The number of cells expressing PD-1 and programmed-death ligand 1 (PD-L1) is low in PC [17]; PD-L1 expression is present in around 30–40% of PC and is associated with severe prognosis [15,16]. In terms of molecular targets, 4–7% of patients with PC have a germline BRCA mutation [18,19,20,21,22]. Olaparib, a PARP inhibitor, has shown good clinical results in patients affected by ovarian or breast cancer and a germline BRCA mutation [23,24]. Therefore, this drug has been tested compared to a placebo in the POLO phase III trial for metastatic PC with BRCA 1–2 germline mutation and without disease progression during the 4 months after first-line platinum-based chemotherapy [25]. The results showed a significantly longer mPFS for the olaparib group, although no benefit was seen for OS [25]. KRAS is another possible predictive biomarker of drug response. It is the most frequently mutated oncogene in cancer; it is a key mediator of the RAS/MAPK signaling cascade that promotes cellular growth and proliferation [26,27,28]. KRAS mutations occur in approximately 90% of PCs, and approximately 2% of these are KRASG12C mutations [29]. Sotorasib and Adagrasib are KRAS G12C inhibitors that have been evaluated in phase II trials (CodeBreak 100 and KRISTAL-1, respectively) for pre-treated metastatic–mutated-PC harboring a KRAS p.G12C mutation, with encouraging results.

Patients with advanced hepatocarcinoma (aHCC) have a poor prognosis despite the new molecular targeted therapies that have been developed in recent years (Sorafenib, Lenvatinib, Cabozantinib, Regorafenib, and Ramucirumab) [30,31]. In consideration of the autoimmune tolerance of the hepatic microenvironment and the chronic inflammation in which HCC often develops, different ICIs have been investigated, both as single agents and combined with other ICIs or antiangiogenetic agents [32]. While single ICI did not demonstrate favorable clinical outcomes, combination therapies demonstrated an important amelioration in survival. In this regard, the combination of Atezolizumab and Bevacizumab has become the new gold standard of treatment. Also, the combination of Durvalumab and Tremelimumab has documented a significant survival improvement and will become a new therapeutic option [33]. However, an important proportion of aHCC patients do not experience a significant efficacy from immunotherapy. Currently, there is still little information about the predictive biomarkers of response to immune-based therapies for aHCC patients. Various studies have investigated different tumor characteristics, such as intact IFN-y signaling, TMB-H, the presence of ICPs, and high levels of Tumor-infiltrating lymphocyte (TILs), which have demonstrated a positive clinical response to immunotherapy [34,35,36,37,38]. Hence, research for find predictive biomarkers is needed with the aim to define which patients can obtain a benefit from a specific therapy. Actually, AFP is the only valuable biomarker for HCC able to guide treatment and predict prognosis [39]. However, other potential biomarkers for HCC have been analyzed such as vitamin K absence-II (PIVKA-II) [40]. Interestingly, some studies put in evidence the advantage of employing multiple biomarkers in concert than a biomarker alone [39]. For example, CRAFITY (C-reactive protein (CRP) and alpha-fetoprotein (AFP) in immunotherapy) is an immunoscore that has been designed for HCC patients to predict prognosis and response to immunotherapies [41]. In addition, the etiology of HCC might be used as a guide for the choice of therapy [42]. In this regard, a meta-analysis showed that immunotherapies may be more effective among patients with viral etiology-related HCC compared to NALFD-related HCC [42].

Biliary tumors are rare, but with a poor prognosis. In recent years, some predictive biomarkers of drug response have been evaluated with interesting efficacy [43]. Pemigatinib, a fibroblast growth factor receptor 2 (FGFR2) inhibitor, was the first targeted agent to be approved for patients with advanced biliary tumors who had FGFR2 gene fusions or rearrangement. More recently, other predictive biomarkers have been identified such as isocitrate dehydrogenase 1 (IDH1), the BRAF V600E mutation, NTRK, and TMB-H/MSI-H/dMMR cancers [44]. Currently, other molecular targets are objects of study including non-BRAFV600E, HER2, and RET mutations [45].

As regards GC, although the recent advances in terms of new efficient drugs, chemotherapy still represents the main treatment option for this tumor. About 20% of GC patients have HER2 overexpression, so the addition of trastuzumab to chemotherapy provides a clinical benefit [46]. Moreover, trastuzumab deruxtecan (T-Dxd) was approved for HER2-positive GC in several countries. In more recent years, immune checkpoint inhibitors (ICIs) also gained a foothold both in the perioperative and advanced setting and in combination with other therapeutic targets including HER2-positive GC [47]. However, several other immune-based therapies including anti-T cell immunoreceptor with immunoglobulin, chimeric antigen receptor T-cell (CAR-T) therapy and immunoreceptor tyrosine-based inhibitory motif domain (anti-TIGIT) therapy have been evaluated [46,47]. Furthermore, alternative therapeutic targets are currently under investigation as predictive biomarkers including claudin 18.2 (25% of GC) and fibroblast growth factor receptor 2b (FGFR2b) (30% of GC) [46].

In conclusion, although many new targeted therapies and therapeutic strategies have appeared for the treatment of GI cancers, they provide a survival benefit only for a small proportion of patients. This happens because just some of GI cancers have a molecular biomarker that can benefit from a targeted therapy. At the same time, immunotherapy is effective only for some GI cancer patients based on the presence of MSI-H. Hence, the clinical need to discover and explore new predictive and prognostic biomarkers in the field of GI cancers with the aim to personalize the management of these patients for individualized care.
